# Building bridges among public health stakeholders in Asia and Europe: the Asia-Europe Foundation (ASEF) Network for Public Health

**Published:** 2011-01-10

**Authors:** Rachmat Irwansjah

**Affiliations:** 1 Asia-Europe Foundation, 31 Heng Mui Keng Terrace, Singapore 119595

## 

The recently occurred pandemic H1N1 has given lessons on the significance of a multisectorial approach in developing mechanism for responses and preparedness against the spread of communicable diseases. This approach can expand beyond the administrative jurisdiction of a country when there is transboundary movement of diseases when measures taken require cross-border coordination. In this notion, regional integration appears to be a mechanism to address this challenge.

The Asia-Europe Foundation (ASEF) Network for Public Health launched in May 2009 was established as part of the wider initiative of Asia-Europe Meeting (ASEM) Foreign Ministers’ Meeting in combating possible human influenza pandemic. Financially supported by the Government of Japan for a period of 5 years (2009-2013), the ASEF Network is part of the wider ASEM Initiative for the Rapid Containment of Pandemic Influenza which also includes a regional stockpile.

The paper will highlight the three thematic working groups of the ASEF Network and their activities for the past two years and how these activities have contributed in building bridges among public health stakeholders in Asia and Europe.

